# Adding to the spectrum of insulin sensitive populations for mixed meal tolerance test glucose reliability assessment

**DOI:** 10.1186/s40200-016-0279-x

**Published:** 2016-12-07

**Authors:** Sabina Paglialunga, Angelica Guerrero, Julie M. Roessig, Paul Rubin, Clayton A. Dehn

**Affiliations:** 1 Celerion, 2420 W Baseline Road, Tempe, AZ 85283 USA; 2 Current Address: Clinical Trials of Texas, San Antonio, TX USA; 3 XOMA Corporation, Berkeley, CA USA; 4 Current Address: Umbrella Corporation, San Antonio, TX USA

**Keywords:** MMTT, Postprandial glucose, Reliability

## Abstract

As a measure of insulin sensitivity, the mixed meal tolerance test (MMTT) is a simple technique that can provide robust results. The assay consists of examining plasma glucose, insulin and C-peptide prior to and following the consumption of a test meal. While this procedure has been used in clinical research for several years, there is no set standard protocol, and only until recently has the reliability of this assay been thoroughly evaluated in prediabetes and type 2 diabetes subjects. Interestingly, the results from this recent study demonstrated stronger MMTT reliability for the prediabetes and diabetes cohorts compared to obese controls. This finding suggests that the obese control group may have more inherent variability in glucose response during a meal challenge likely due to compensatory influences typically observed in non-diabetic insulin-resistant subjects. Furthermore, this study raises the question whether the MMTT assay is reliable in a non-obese cohort. Therefore, to promote the standardization of this technique and contribute to the band of insulin sensitive populations, we employed the same methodology and test meal as the reference study to evaluate the MMTT reliability in healthy and overweight men. Indeed, the interclass coefficient revealed high glucose response repeatability during the MMTT in insulin-sensitive men. Overall, the MMTT is a reliable test across a range of insulin sensitivity including healthy men. However, we propose further investigation may be required to fully define the utility of this methodology in obese non-diabetic insulin-resistant populations.

## Background

A mixed meal tolerance test (MMTT) is a technique that evaluates insulin sensitivity and β-cell function through computational indices. Typically, fasting and postprandial glucose, insulin and C-peptide serial blood samples are collected 2–4 h after the consumption a test meal. Although routinely used in clinical research, there is no operational standard regarding sampling interval, assay duration, or test meal composition. To standardize this methodology, the Foundation for the National Institutes of Health (FNIH) Biomarkers Consortium recently examined the reliability of a MMTT consisting of a nutritional drink and supplement bar (BOOST® and PowerBar® respectively, Nestlé Health Science, NJ) in subjects with varying degrees of insulin resistance [1]. Reproducibility of the MMTT was evaluated in a test-retest study of obese subjects with normal glucose tolerance (NGT), prediabetes or type 2 diabetes. Intriguingly, the FNIH observed stronger reliability in glucose area-under-the-curve (AUC) for the prediabetes (ICC = 0.87) and type 2 diabetes (ICC = 0.73) cohorts over the NGT group (ICC = 0.39) [1], where an interclass correlation coefficient (ICC) >0.75 indicates a highly repeatable assay [2].

## Examining MMTT reliability in healthy and overweight men

Contributing to the investigation of various populations along the spectrum of insulin sensitivity, we examined the reliability of a MMTT in healthy and overweight males. While Short et al. previously reported high MMTT reproducibility of a liquid test meal administered to healthy subjects [3], we employed the same test meal and operational techniques as the FNIH [1]. Since increasing study number observations can improve ICC precision [4], four men were recruited to undergo five-serial MMTTs over the course of one week.

## Study design

Healthy adult males 18–45 years old, with BMI values between 18–29 kg/m^2^, fasting glucose <100 mg/dL, and HbA1c ≤5.6% were recruited to take part in a clinical study examining glucose response to a mixed meal tolerance test. The study protocol was approved by an ethics research board and written informed consent was obtained from each subject. The results were obtained from a sub-analysis of a larger double-blind, placebo-controlled clinical study. The current analysis includes subjects on the placebo dose only. Four subjects repeated the MMTT on 5 different days (Day 1, 2, 3, 4, and 7) over the span of 1 week. Participants were housed at the clinic over the course of the study week. Standardized meals for a total of ~2200 calories (10–35% protein, 20–35% fat and ~45% carbohydrate) per day were provided for breakfast, lunch and dinner. On the experimental days, breakfast was replaced with the MMTT and was performed as described by Shankar et al. [1]. Briefly, subjects were fasted for 12 h prior to the MMTT. A test meal containing 1 BOOST® drink and 1 PowerBar® (470 kcal; 71 g carbohydrate, 8.5 g fat, 20 g protein, Nestlé Health Science, NJ) was given at breakfast (~8:30 am), and subjects were instructed to consume the entire meal within 15 min. Serial blood draws were taken just prior (~0) and 15, 30, 60, 90, 120, 210, and 240 min following the start of the meal. Plasma glucose was measured on a YSI 2300 STAT PLUS^TM^ (Yellow Springs, OH). Fasting and Day 2 plasma insulin was measured by Cobas® (Roche Diagnostics Limited, Switzerland), according to manufacturer’s instructions.

## MMTT is highly repeatable in insulin sensitive men

**Fig. 1:**
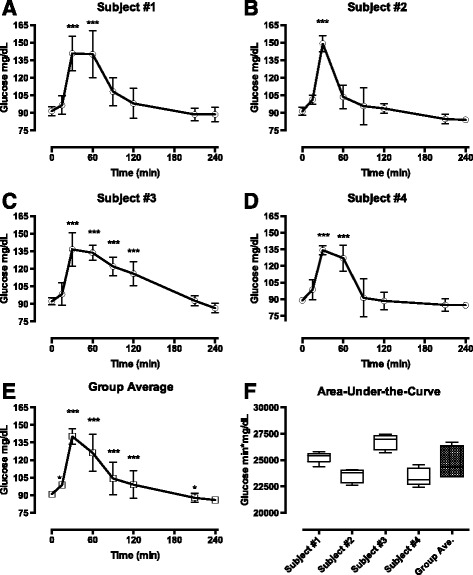
Average glucose response to a MMTT. **a-d** Average plasma glucose during the MMTT over five test days from four subjects. Results presented as mean ± SD, *n* = 5. **e** Group average glucose curve presented as mean ± SD, *n* = 4. Glucose curves were analyzed by two-way ANOVA followed by Dunnett’s post-hoc test where *p < 0.05, **p < 0.01, ***p < 0.001 vs. time 0. No one day was significantly different from another. **f** Box and whisker plot of average area-under-the-curve (AUC) for subjects 1 to 4 and group average. Group AUC demonstrated low within-subject (2–4%) and between-subject (6%) variation as determined by percent coefficient of variation

Baseline characteristics of the cohort are displayed in Table 1. Overall, these men were younger with a lower BMI, greater insulin sensitivity, and lower fasting insulin, then those in the FNIH NGT group [1]. Glucose curves (Fig. 1) and AUC was very repeatable (ICC [95% confidence interval] = 0.83 [0.50,0.99]), consistent with previous liquid MMTT results in healthy subjects (ICC = 0.87) [3]. The evidence for the MMTT as a reliable testing method in healthy, prediabetes and type 2 diabetes populations is compelling, however the lack of repeatability in obese NGT subjects is puzzling and worthy of further exploration.

**Table 1:** Cohort baseline characteristics

Parameter	Average ± SD	Range (min;max)
Age (years)	30 ± 8.9	22;40
Body weight (kg)	175.4 ± 14.4	158.0;190.4
Body mass index (kg/m^2^)	26.0 ± 2.9	23.0;28.8
Fasting glucose (mg/dL)	90.3 ± 0.8	89.2;91.0
Fasting insulin (μIU/mL)	7.6 ± 1.0	6.5;8.5
HbA1c (%)	4.9 ± 0.3	4.5;5.3
HOMA-IR	1.7 ± 0.2	1.4;1.9
Insulin Total AUC 0–240 min (μIU/mL*min)	9649.9 ± 3313.6	5399.3;13494.0

Plasma values obtained from experimental Day 1. Insulin total AUC was measured on Day 2

Interestingly, the FNIH NGT group displayed traits associated with greater risk for underlying insulin resistance such as fasting insulin >10 μIU/mL [1], which was higher than values observed here. Although glycemia in the NGT group may have been within normal limits, this control was likely facilitated through mild compensatory hyperinsulinemia. Four-hour insulin AUC for the NGT group was ~12500 μIU/mL*min [5], which is approximately 29% higher than obtained during the present study in healthy and overweight men (Table 1). Therefore, the NGT group may be more accurately described as Non-Diabetic Insulin-Resistant (NDIR) [5]. Since hyperglycemia, the clinical hallmark of diabetes, in this population is masked by a progressively failing compensatory insulin response, the variability observed within this intermediate metabolic state could be quite profound and impactful on the reliability of the MMTT. The repeatability of the MMTT glucose parameter within the NGT/NDIR group might be substantially improved by intensifying qualification criteria to select for a more homogenous population.

## Study limitations

There are several limitations of the current study which must be addressed. Due to the sub-analysis nature of this study, there were restrictions in study design considerations and sample analysis. The inclusion criterion of healthy and overweight men only limits the analysis to a relatively fit population; glycemic excursions can be more variable in obese states, and we cannot address gender effects. Although, others have shown adjusting for sex does not impact MMTT results [6]. Also, aside from Day 2 insulin response, we did not measure insulin and C-peptide throughout the MMTT experiments, and rely on our glucose data as a main outcome. Finally, our analysis consisted of only four individuals. However, despite the small sample size, we were able to show strong reproducibility in the glucose response. Moreover, since the ICC calculation is dependent on sample size [7], with such low intra-subject variation in glucose response, a larger cohort may have inflated our ICC score.

## Conclusion

The ultimate goal of a standardized MMTT is to examine insulin sensitivity with a simple, non-invasive assay. Akin to the OGTT, which has set cut-off values to define glucose tolerance, with further research a standardized MMTT holds the potential to stage insulin sensitivity based on predefined ranges. Altogether, the MMTT is a suitable, reliable test across the spectrum of insulin sensitivity including healthy and overweight men. However, further investigation may be required to fully define the utility of this methodology in NGT/NDIR populations.

## Abbreviations

AUC: Area-under-the-curve
BMI: Body mass index
FNIH: Foundation for the National Institutes of Health
ICC: Interclass coefficient
MMTT: Mixed meal tolerance test
NDIR: Non-diabetic insulin-resistant
NGT: Normal glucose tolerance
OGTT: Oral glucose tolerance test
